# The Impact of Phenocopy on the Genetic Analysis of Complex Traits

**DOI:** 10.1371/journal.pone.0011876

**Published:** 2010-07-29

**Authors:** Francesco Lescai, Claudio Franceschi

**Affiliations:** 1 Division of Research Strategy, University College London, London, United Kingdom; 2 Centre “L. Galvani” for Biocomplexity, Alma Mater Studiorum - Università di Bologna, Bologna, Italy; 3 Department of Experimental Pathology, Alma Mater Studiorum - Università di Bologna, Bologna Italy; GSF Research Center for Environment and Health, Germany

## Abstract

A consistent debate is ongoing on genome-wide association studies (GWAs). A key point is the capability to identify low-penetrance variations across the human genome. Among the phenomena reducing the power of these analyses, phenocopy level (PE) hampers very seriously the investigation of complex diseases, as well known in neurological disorders, cancer, and likely of primary importance in human ageing. PE seems to be the norm, rather than the exception, especially when considering the role of epigenetics and environmental factors towards phenotype. Despite some attempts, no recognized solution has been proposed, particularly to estimate the effects of phenocopies on the study planning or its analysis design. We present a simulation, where we attempt to define more precisely how phenocopy impacts on different analytical methods under different scenarios. With our approach the critical role of phenocopy emerges, and the more the PE level increases the more the initial difficulty in detecting gene-gene interactions is amplified. In particular, our results show that strong main effects are not hampered by the presence of an increasing amount of phenocopy in the study sample, despite progressively reducing the significance of the association, if the study is sufficiently powered. On the opposite, when purely epistatic effects are simulated, the capability of identifying the association depends on several parameters, such as the strength of the interaction between the polymorphic variants, the penetrance of the polymorphism and the alleles (minor or major) which produce the combined effect and their frequency in the population. We conclude that the neglect of the possible presence of phenocopies in complex traits heavily affects the analysis of their genetic data.

## Introduction

Highthroughput genetic analysis represents the present and the future in catching the genetic determinants of complex diseases[1], [2], [3], [4], [5], [6]. A consistent debate is ongoing on the best approaches to overcome the major issues inherent to genome-wide association (GWA) study designs[7], [8], [9], [10], [11], [12], [13], [14], [15], [16], [17], [18].

The most widely used statistical tests are single point statistics (chi-square, or Cochrane-Armitage test) along the genome; these tests can be integrated with haplotype (or multi-marker) analysis once the linkage disequilibrium (LD) structure is drawn and thus haplotype blocks have been identified.

All these tests can be performed under different assumptions and with slightly different approaches, and multivariate analyses are generally performed.

Two main obstacles can be envisaged as:

1. the false positive rates, and consequently the efficacy of the corrections adopted;
2. the capability to identify low-penetrance variations across the human genome.

As for false positives, many different approaches have been proposed and, provided the sample collection to be large enough, a multi-stage design has been shown to be very effective in detecting key leads in the genome, often replicated in other populations. It's not the purpose of this paper to address this area[7], [19].

As for the identification of low-penetrance polymorphisms, the area is of a major consideration when disentangling the picture of any complex trait. Indeed, it's quite realistic for complex phenotypes to be determined by a combination of many different polymorphic loci each of them accounting for a minor part of the total variance[20], hence very difficult to be detected when a genome-wide genotyping is performed and when GWA significance rates are applied[20].

Despite this issue being of a key importance, most of the papers reporting GWA studies applied single point statistics, multi-marker analysis and haplotypes analyses, performed LD mapping, adopted different false-positive rate corrections[21], [22], [23], [24], [25]. Few of them actually included interaction analysis and other similar approaches capable to grasp the effect of interactions and across-genome combinations, rather then the main effect of single markers or (despite more importantly) the major contribution of a specific haplotype in a locus[26], [27], [28].

Among the phenomena reducing the power of these analysis, phenocopy hampers very seriously the investigation of complex diseases, a well known issue in neurological disorders [29], [30], cancer [31], and likely of primary importance in the study of human ageing [32]. However, the concept of phenocopy is quite old in genetics, and assumed different meanings according to many different authors: for the purpose of this paper, we mainly refer a definition adopted in linkage studies, where “phenocopy” indicates affected individuals who had acquired the disease by different means than the ones segregating in rest of the family[33]. Moreover, the term here needs to be even more focused, due to the characteristics of the simulating algorithm adopted in this study to generate the disease model and subsequently the datasets: globally we consider here a “phenocopy” an individual marked as affected, but where the underlying genetic markers associated with the disease are different from the other cases in the dataset. We also aknowledge that the classical definition of phenocopy assumes a smooth and wider perspective when we consider the most important complex traits: in this scenario its importance appears to be even higher, due to its intrinsic presence when the interplay of multiple genetic loci determines a disease. Phenocopy (indicated as PE, “phenocopy error”, from the terminology of the genomeSIMLA software) seems to be the norm, rather than the exception, especially when considering the role that epigenetics and environmental factors exert on the phenotype [34].

Considering the scenario we are dealing with, additional terminology needs to be clarified. As previously mentioned, one of the hot topics geneticists are currently debating is whether the so called “missing heritability” issue would find an answer in very rare and highly penetrant mutations (detectable with exome sequencing or whole genome next generation sequencing only [35]), or in a multitude of polymorphisms with no effect when considered alone (main effect) but with a more significant effect when their statistical interaction is considered [36], [37].

As far as this latter point is concerned, several models have been proposed since many years [38] which define “epistasis” (again another term used with different meanings in genetics) as the interaction between different loci, and call “purely epistatic” those interactions between loci that do not display any single locus main effect [37], [38], [39]. This model has been proposed and largely debated [34], [40], [41]: some authors consider the additive model widely used as sufficient to incorporate these effects[42], or argue about the scarce impact of such a scenario, but few papers address specifically this topic[43], [44].

Despite some attempt [45], [46], [47], no widely recognized solution has been therefore proposed, particularly to estimate the effects that phenocopies could exert either on the study planning or its analysis design. At present, the most of the analysis strategies do not take into account the intrinsic presence of phenocopy in complex traits.

We present a simulation [48], [49], [50], [51], where we attempt to define more precisely how phenocopy impacts on different analytical methods under different scenarios.

## Results

### Simulation of the datasets

Two disease models have been simulated.

In the first model, i.e “model ME”, standing for “Main Effect”, the marker RL0-855 was simulated, having a main effect and an OR = 2.225. Three additional SNPs (Table 1) have been simulated with a very small marginal effect, and an interaction associated with the disease, according to the mixed model offered by the logistic function of genomeSIMLA.

**Table 1:** The table summarizes the characteristics of the genetic model implemented in the ME model, where one SNP with main effect has been simulated.

Main effect SNP			
dataset		target β	target OR
**main dataset**	**RL0-855**	**0,80**	**2,225540928**
additional 01	RL0-179	0,80	2,225540928
additional 02	RL0-111	0,80	2,225540928
additional 03	RL0-210	0,80	2,225540928
additional 04	RL0-503	0,80	2,225540928
additional 05	RL0-995	0,80	2,225540928

Interacting SNPs			
		main effect β	target OR
RL0-75		0,000000001	1,000000001
RL0-245		0,000000001	1,000000001
RL0-457		0,000000001	1,000000001

The additional datasets used to pick-up the phenocopies, as implemented in the PM2 method are indicated.

In the second model, i.e. “model EPI”, standing for “purely epistatic”, the second disease model (model EPI), three markers (RL0-75 RL0-153 and RL0-272, Table 2) have been simulated in order not to display any main effect and associate with the disease with a purely epistatic penetrance table, with target OR = 4.

**Table 2:** The table summarizes the SNPs modelled in the purely epistatic model generation, whose penetrance function target odds ratio was set to 4.

Epistatic only alternative datasets		
dataset	interacting SNPs	target OR
**main dataset**	**RL0-75 RL0-153 RL0-272**	**4**
additional 01	RL0-66 RL0-155 RL0-268	4
additional 02	RL0-123 RL0-79 RL0-337	4
additional 03	RL0-63 RL0-125 RL0-332	4
additional 04	RL0-66 RL0-116 RL0-292	4
additional 05	RL0-63 RL0-120 RL0-329	4

The additional rows indicate the SNPs modelled in the additional datasets used to pick-up phenocopies according to the PM2 method for phenocopy generation.

For each disease model, the following datasets have been extracted from the population: a) 6 different case-control datasets with increasing phenocopy level generated with the method implemented within the software (PM1); b) 6 different case-control datasets with increasing phenocopy level generated with an alternative method (PM2) develop in our lab, as described in materials and methods; c) 6 pedigree datasets with increasing phenocopy level generated as implemented in genomeSIMLA.

### Main effect model

**Figure 1:**
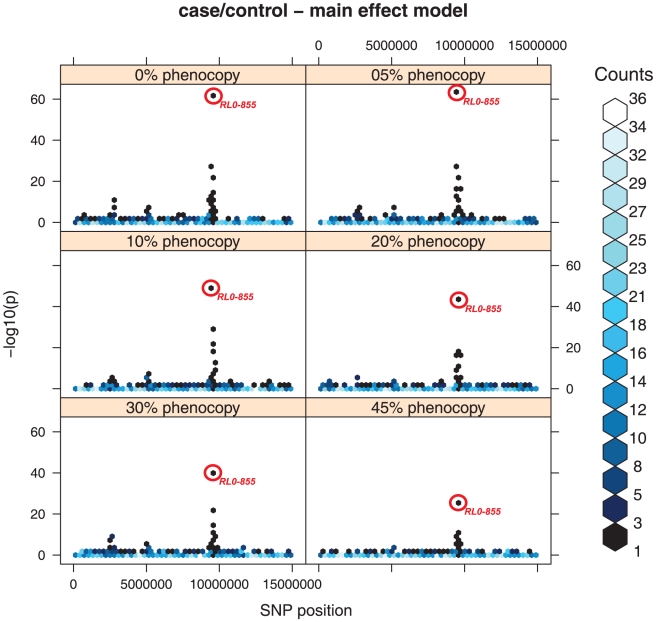
Case/control dataset - main effect model. Single point association analysis of the chromosome where a mixed model of marginal effects and interactions were simulated. The picture shows the impact of the different levels of phenocopy error (PE) on the significance levels. The point surrounded with a red circle indicates the bin where the SNP with a main effect is located, showing its significance level in the single point association analysis. In order to simplify the plot, groups of SNPs with similar p values have been grouped into “bins”, as performed by the *hexbin* package in R. The number of markers in each bin is represented by different shades of blue, as indicated in the legend.

As far as the model ME is concerned, the results show that strong main effects are not hampered by higher levels of PE, despite an inflation of the significance (figure 1).

In the case-control dataset with PM1 method, RL0-855 was highly significant at each phenocopy level until 45%, displayed a −log10(p) = 62.54 at 0%PE and a −log10(p) = 25.84 at 45%. The analysis of the datasets obtained with the PM2 phenocopy algorithm produced similar results (see Supplementary Figure S1): the RL0-855 was significant in the 0% phenocopy dataset with a −log10(p)  = 67.5, and a −log10(p)  = 31.2 in the 45% dataset.

A very similar behaviour appears to happen on the pedigrees dataset, with TDT analysis, even if the overall significance level is a bit lower (−log10(p) = 40 at 0%PE and −log10(p) = 8.63, see Supplementary Figure S2).

Among the other markers where only an interaction was simulated, only the marker RL0-245 appeared among the top ten significant at 0%PE (−log10(p) = 11.47) but it was no more on the top 10 when the phenocopy level reached 10%. The same happened on the TDT analysis.

### Purely epistatic model

When we analyzed the EPI model on the case control dataset, none of the three markers ranked among the top list of significant markers. Moreover if we had to correct for multiple testing, none of the markers would reach a 0.05 level of significance neither at 0% PE level, nor at 45%.

**Figure 2:**
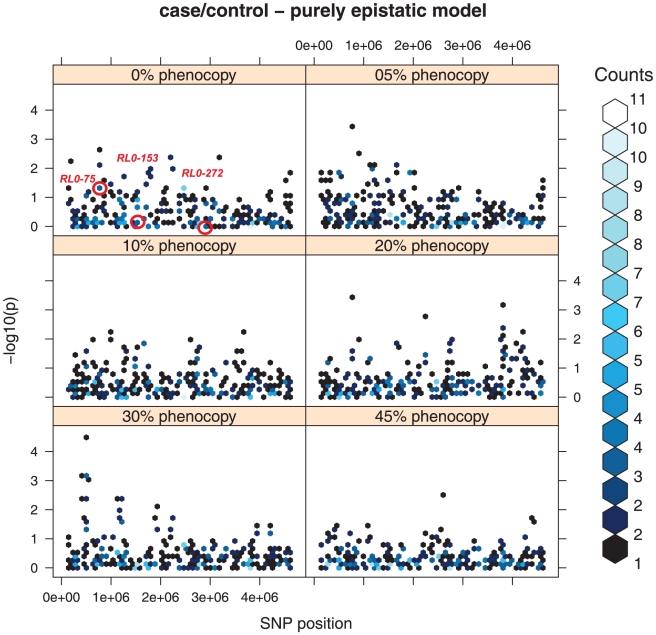
Case/control dataset - purely epistatic model. Single point association analysis of the chromosome where only purely epistatic effects were simulated. The picture shows the impact of the different levels of phenocopy error (PE) on the significance levels. The points surrounded with a red circle indicates the bin where the three interacting SNPs are located, showing their significance level in this single point association analysis. In order to simplify the plot, groups of SNPs with similar p values have been grouped into “bins”, as performed by the *hexbin* package in R. The number of markers in each bin is represented by different shades of blue, as indicated in the legend.

Despite some fluctuations on the data, mainly due to sampling and data extraction, a positive but no significant trend in the number of falsely significant markers could be observed according to the increase of phenocopy error percentage (figure 2). The same pattern was observable when analyzing the case-control dataset generated with the PM2 phenocopy method (see Supplementary Figure S3).

When applying PM2 we observed the appearence of a single progressively significant marker (RL0-255), which was borderline for the Hardy-Weinberg equilibrium in the main dataset and therefore was unbalanced when affected individuals from different dataset suffering the same simulation phenomenon were added. This SNP can be considered a false positive, as it was not simulated in association of the disease in none of the additional datasets.

A similar behaviour of the markers with a purely epistatic effect was observable in the pedigree dataset with a TDT analysis: again none of them ranked as significant (Supplementary Figure S4).

In order to check for the correctness of the model we generated, we performed a logistic regression on the interaction term between the three markers we simulated to be associated with a purely epistatic effect. The p value of the logistic regression was highly significant both at a 0% PE (p = 7.8*10^−21^) and at a 45% PE (p = 4.17*10^−6^).

**Figure 3:**
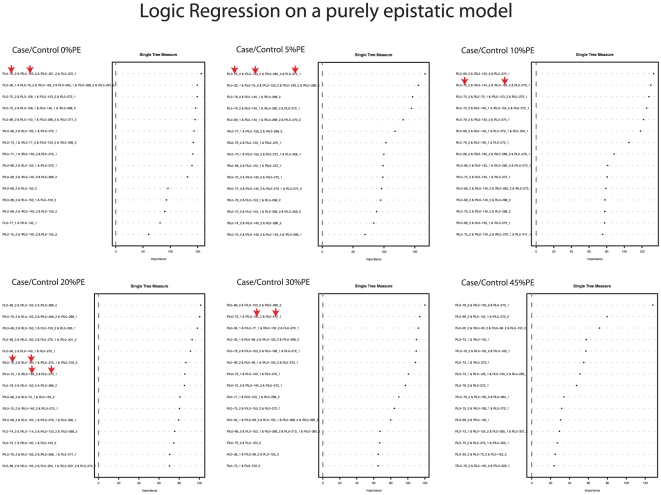
Logic regression on a purely epistatic model. This plot is generated for each dataset of case-control simulations by using a “logic regression” approaches, and shows the rank of importance of the interactions identified. By applying this methodology the analysis was capable to identify in most cases two of the three interacting SNPs among the top ranking interactions. The captured associates SNPs are highlighted by arrows.

Therefore we decided to analyze the data by using a logic regression approach. Logic Regression is an adaptive regression methodology mainly developed to explore high-order interactions in genomic data and its goal is to find predictors that are Boolean (logical) combinations of the original predictors. By applying this methodology the analysis was capable to identify in most cases two of the three interacting SNPs among the top ranking interactions (figure 3).

The more the phenocopy error was increasing and the more these interactions ranked lower, even if in any case at least one of the three markers (RL0-153) was always present among the top five.

As a purely epistatic model is a challenge for the analysis in itself, we adopted a further analysis method, i.e. the multifactor dimensionality reduction (MDR)[44], [52]. MDR analysis was performed on the EPI model with PM2 phenocopy levels.

Comparably with the logic regression analysis, the MDR method perfomed with random non exhaustive explorations, was unable to catch efficiently all the interactions, and this became more evident with increasing PE levels (Supplementary Table S1). When testing directly the interacting SNPs, the efficiency and the OR of the MDR outcome was very close to the modelled one, but these values progressively decreased the more the PE level increased: at a 0% PE the predicted OR was 3.80 (compared to a target OR of the model = 4.0) and at 45% PE the predicted OR decreased to 2.39 (table 3, and Supplementary Figure S5 and Supplementary References S1).

**Table 3:** MDR test on purely epistatic model interactions.

	Phenocopy level					
	0%	5%	10%	20%	30%	45%
Testing Accuracy	0.6597	0.6539	0.6492	0.6303	0.6271	0.6098
Testing Sensitivity	0.7061	0.7473	0.7009	0.682	0.5963	0.5655
Testing Specificity	0.6132	0.5621	0.5993	0.5824	0.6547	0.6474
Testing Odds Ratio (CI)	**3.8087** (3.1613,4.5887)	**3.7956** (3.1397,4.5885)	**3.5049** (2.9117,4.2188)	**2.9913** (2.4901,3.5934)	**2.8012** (2.3359,3.3591)	**2.3893** (1.9947,2.862)

This table summarizes the test performed with the multifactor dimensionality reduction method on the RL0-75/RL0-153/RL0-272 interactions at different phenocopy levels generated with the PM2 method. The target odds ratio for the purely epistatic model of the original dataset was 4: it is evident how at increasing level of phenocopy, the odds ratio captured by the test for the interacting SNPs progressively decreases.

## Discussion

Investigating the genetic determinants of complex traits challenges researchers with obstacles yet unresolved completely. We can argue that the genetic scenario of the most important complex traits is not explainable in black and white, i.e. only by the presence of very rare variants yet to be discovered with sequencing or by the presence of purely epistatic effects. Complex traits are likely determined by a different contribution of both causes, with proportions that can differ from a phenotype to another. In this paper we chose to address this second aspect which deserves specific attention.

The characterization of the phenotypes is of extreme importance to this regard, and in our work we focused simulations of genetic data on the analysis of the effect that phenocopy levels could have in the capability to understand the genetic determinant of a disease with different methodologies.

We would like to stress that the concept of “phenocopy” can be interpreted in several ways, as we pointed out in the introduction, and that the classical definitions of phenocopies should be largely revisited in the context of complex traits, where multilocus genotypes could play a decisive role. Yet this aspect plays a major role in the discovery of genetic determinants: if to a certain extent complex traits could be considered by definition phenocopies, and if purely epistatic interactions play an important role in the missing heritability (perhaps along undiscovered rare variants), then future analysis methods have to take into account this scenario and model not only interactions, but also phenocopy within their statistical model.

In our simulation we decided to verify the impact of phenocopy level by testing two methods for the generation of phenocopies: the PM2 method we developed, specifically produces phenocopies by introducing affected individuals in which different genetic determinants have been simulated. The PM2 method thus allowed us to test a scenario where different combinations of loci could produce the same phenotype.

Our results show that strong main effects are not hampered by the presence of an increasing amount of phenocopy in the study sample, despite progressively reducing the significance of the association, if the study is sufficiently powered.

On the opposite, when purely epistatic effects are simulated, the capability of identifying the association depends on several parameters, such as the strength of the interaction between the polymorphic variants, the penetrance of the polymorphism, the alleles (minor or major) which produce the combined effect and their frequency in the population. The influence of these parameters has been partially discussed in 0% PE datasets in the literature. In our simulation the critical role of phenocopy emerges, and the more the PE level increases the more the initial difficulty in detecting these gene-gene interactions is amplified, even with methodologies more suitable to the discovery of epistatic models.

Classical analytical methodologies are very sensible to this error, and new statistical methods have to be developed, addressing in a less computing-intensive way SNP-SNP interactions as well as accounting or adjusting their results on estimates of the phenocopy error.

Since the presence of phenocopy can be a characteristic intrinsic to the phenotyping of complex traits, we conclude that the neglect of the possible presence of phenocopies in these scenarios heavily affects the analysis of their genetic data.

## Materials and Methods

### Simulations

We performed simulations by using the software genomeSIMLA[50] which performs the simulation of large-scale genomic data both in population based case-control samples and in families. It is a forward-time population simulation algorithm that allows the user to specify many evolutionary parameters and control evolutionary processes and allows the user to specify varying levels of both linkage and LD among and between markers and disease loci. [48], [49], [53]. Particular SNPs may be chosen to represent disease loci according to desired location, correlation with nearby SNPs, and allele frequency. Up to six loci may be selected for main effects and all possible 2 and 3-way interactions. Disease-susceptibility effects of multiple genetic variables can be modeled using either the SIMLA logistic function [49], [53] or a purely epistatic multi-locus penetrance function [41] found using a genetic algorithm to assign affected status (for program configuration files see Supplementary Model S1).

### Disease models

We generated two different disease models.

**Figure 4:**
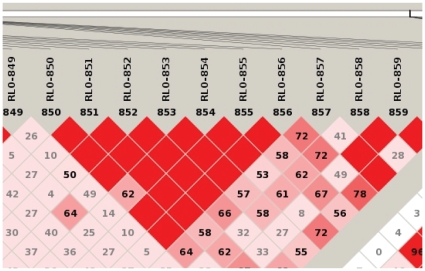
LD plot from main effect model dataset. Linkage disequilibrium plot of a small portion of the simulated chromosome in the dataset with main effects. The LD block where the associated SNP (RL0-855) is simulated is visible in the picture. The existence of a block encompassing seven markers also explains the signal associated with other few SNPs in strong LD with RL0-855.

In the first one (referred to as “model ME”, standing for “Main Effect”) a single SNP (RL0-855, figure 4) was simulated to have a main effect on disease, with an OR = 2.225; at the same time the disease model included also three other SNPs (RL0-75, RL0-245, RL0-457) with no main effect and an interaction associated to the affection status. We simulated this model on a single chromosome with 1.362 markers.

In the second model (referred to as “model EPI”, standing for “purely epistatic”), we performed a simulation on a smaller chromosome (401 markers), where no main effect was present and three SNPs (RL0-75, RL0-153, RL0-272) were affecting disease with only a purely epistatic disease model, generated by using SIMPEN [49]. The penetrance table was generated with a target OR = 4.

In both simulations the SNP chosen to be associated with the disease had a MAF>0.30, in order to allow us to simulate the condition so called “common variant common disease”[54], [55], [56]. Table 1 and Table 2 provide information on the associated markers and their target OR. Supplementary Figure S6 gives additional details on the disease model generation.

For each of the two models case-control data and pedigree data were generated. On each case six different large pooled datasets were extracted, with an increasing level of phenocopy error (i.e. 0%, 5%, 10%, 20%, 30% and 45%). In order to avoid biases due to data extraction and fluctuation, each dataset has been obtained by sampling and then pooling 50 different datasets on each PE level.

The case/control simulation included datasets of 200 cases and 200 controls each, i.e. finally 20.000 individuals each PE level dataset.

Each family simulation included 25 families with 1 affected sib and 2 unaffected, 25 families with 3 affected and 1 unaffected, 25 families with 2 affected, 2 unaffected sibs and 3 random extra sibs: the total number of individuals for each dataset of different PE level was 25.000 samples. Supplementary Figure S7 gives additional details on the datasets generation.

### Generation of the phenocopies

The genomeSIMLA software version used (1.0.7w32), currently implements a method for generating the phenocopy designed as follows.

The software generates cases and controls using the penetrance function and the marker specified by the user. Then, in case-control datasets, it removes a percentage (user specified) of cases and replace them with individuals sampled from the control individuals in the full population and assign them the affected status. In family datasets, the software determines the total number of affected to modify as phenocopies, identifies the pedigrees to be modified and redraw the family according to the new requirements. Pedigrees with the required number of affected and unaffected are selected and then the unaffected phenocopies are marked as affected, according to the initial design specified by the user (personal communication).

This method has been referred as “phenocopy method one” (PM1).

In order to verify the correspondence of such phenocopy generation method with what we defined as “phenocopy” (see introduction), we also developed another methodology to be applied on the case-control datasets only. According to this second algorithm (referred into the article as “phenocopy method two”, PM2), five additional datasets have been generated, with different markers associated to the affected status. In order to generate the phenocopy level required, a uniform random sampling of affected individuals from the five additional datasets have been performed, and these individuals have been substituted with affected individuals randomly picked up from the original dataset. This method generates five datasets with the same phenocopy percentage as the PM1. Supplementary Figure S8 provides a more detailed explanation and supplementary Box S1 reports the R code used to generate these datasets. Table 2 provides information about the markers associated to the affection status in the additional datasets and the target OR used.

### Statistical analysis

The analysis were conducted using the R software (www.r-project.org) and PLINK. In particular whole-chromosome case-control analysis and TDT analysis were performed with PLINK and visualized with R. The calculation of genetic contrasts and the logistic regression on single markers, markers' interaction analysis with logistic regression where performed according to Clayton as developed in the “DGCgenetics” package. Interaction analysis by using a logic regression approach was performed by using the R package “logicFS” by Schwender, according to the developer's specifications.[27]

The MDR analysis has been conducted by using the MDR java package (www.epistasis.org)[57] and performing 5.000 random explorations in the model discovery of attributes ranging from 2 to 4-way interactions, as implemented in the software.

## Supporting Information

Model S1 Model Configuration files.
(0.01 MB ZIP)

Box S1 R code used to generate the alternative phenocopy method datasets.
(0.03 MB DOC)

References S1 References cited in the Supplementary Information.
(0.03 MB DOC)

Table S1 The table summarizes the 10 best models for each phenocopy level identified during the MDR analysis. It has to be stressed that the MDR analysis has been conducted by performing 5.000 evaluations of possible interactions. An exhaustive analysis as implemented in the software would be computationally very intensive, as pointed out by the authors in a recent paper (see Pattin K. A. et al. [4]). In bold the correct SNPs as modelled in the purely epistatic penetrance function.
(0.08 MB DOC)

Figure S1 For the case-control dataset generated with the main effect disease model (see SF6), an alternative method of producing phenocopies has been applied (see SF8). The method displays the same performance of the internally implemented one, with the only exception of few markers which progressely fall outside the equilibrium of Hardy-Weinberg, thus resulting in a false-positive association (indicated by the arrow). The red circle indicates the marker associated with the disease in the main dataset.
(1.29 MB EPS)

Figure S2 The figure summarizes the significance level for each marker in the pedigree datasets simulated with a main effect disease model at each phenocopy level. The red circle indicates the marker associated with a main effect to the disease in the model. The PM1 phenocopy generation method was applied.
(1.31 MB EPS)

Figure S3 For the case-control dataset generated with the purely epistatic disease model (see SF6), an alternative method of producing phenocopies has been applied (see SF8). The method displays the same performance of the internally implemented one, with the only exception of one marker which progressively falls outside the equilibrium of Hardy-Weinberg, thus resulting in a false-positive association (indicated by the arrow).
(1.22 MB EPS)

Figure S4 The figure summarizes the significance level of the markers in pedigree datasets, at each phenocopy level. The red circles indicate the position of the markers associated in the model, which is the same in the other plots.
(1.54 MB EPS)

Figure S5 MDR attribute construction. The figures illustrates the distribution of cases (left bars) and controls (right bars) when the three associated SNPs are considered jointly.
(2.07 MB EPS)

Figure S6 Two disease models have been applied. In the first model a single SNP displays a main effect (target OR = 2.225) and three additional SNPs do not have a main effect and interact with each other with a modest effect; this model is implemented as part of the SIMLA logistic function[1]. In the second model instead, three SNPs have been simulated as having no main effect, and a purely espistatic effect on the disease (with a target OR = 4); this model has been implemented in genomeSIMLA and it has been proposed by Culverhouse [2] and discussed by Moore [2], [3].
(1.07 MB EPS)

Figure S7 For each disease model, two groups of datasets have been generated: a case-control dataset and a family based dataset. In order to reduce the fluctuations due to the sampling, in each case 50 different smaller datasets have been independently sampled from the population and then merged together in order to obtain a large pooled dataset. The figure explains the process step by step.
(1.37 MB EPS)

Figure S8 The method has been developed by using the R software (code provided) in order to perform a random sampling from five additional datasets where different SNPs have been associated in the disease model with the affected individuals. A uniform and random sampling, followed by a random substitution of the individuals in the original dataset produced different levels of phenocopies in the sample, thus generating six dataset with increasing phenocopy percentage. This method ensures the effective substitution of individuals generated as affected but with completely different causative markers. The method has been developed as a further analysis of possible effect generated by the “phenocopying” method implemented in the genomeSIMLA software.
(1.67 MB EPS)
