# Supplementary material for: The Impact of Phenocopy on the Genetic Analysis of Complex Traits
Source: PLoS One. 2010 Jul 29;5(7):e11876. doi: 10.1371/journal.pone.0011876 (PMC2912380; doi:10.1371/journal.pone.0011876)
Supplement: Box S1 — R code used to generate the alternative phenocopy method datasets. (0.03 MB DOC) [file pone.0011876.s002.doc]

**Supplementary Box S1 – R code used to generate the phenocopies**

#author: Francesco Lescai

#contact: f.lescai@ucl.ac.uk

#

#this reads the different pedfiles

original<-read.table("case-control.original.ped",header=F,as.is=T)

case01<-read.table("case-control.case01.ped",header=F,as.is=T)

case02<-read.table("case-control.case02.ped",header=F,as.is=T)

case03<-read.table("case-control.case03.ped",header=F,as.is=T)

case04<-read.table("case-control.case04.ped",header=F,as.is=T)

case05<-read.table("case-control.case05.ped",header=F,as.is=T)

#

#subsetting cases and controls

original.case<-subset(original, original[[6]]==2)

original.control<-subset(original, original[[6]]==1)

case01.case<-subset(case01, case01[[6]]==2)

case02.case<-subset(case02, case01[[6]]==2)

case03.case<-subset(case03, case01[[6]]==2)

case04.case<-subset(case04, case01[[6]]==2)

case05.case<-subset(case05, case01[[6]]==2)

#

#creates the numbers of individuals to be phenocopied

pe05<-length(original.case[[1]])/100*5

pe10<-length(original.case[[1]])/100*10

pe20<-length(original.case[[1]])/100*20

pe30<-length(original.case[[1]])/100*30

pe45<-length(original.case[[1]])/100*45

#

#extraction vectors from each dataset

phenovalue<-c(pe05,pe10,pe20,pe30,pe45)

datasets<-list(case01.case,case02.case,case03.case,case04.case,case05.case)

for (i in 1:length(phenovalue)) {

newcase<-original.case

phenodata<-data.frame()

for (d in 1:length(datasets)) {

pheno<-datasets[[d]]

randomchoice<-floor(runif(n=phenovalue[i],min=1,max=length(case01.case[[1]])))

randomchoice<-unique(randomchoice)

randomchoice<-randomchoice[(1:(phenovalue[i]/5))]

pheno<-pheno[randomchoice,]

phenodata<-rbind(phenodata,pheno)

}

randompheno<-floor(runif(n=phenovalue[i]*2,min=1,max=length(original.case[[1]])))

randompheno<-unique(randompheno)

randompheno<-randompheno[(1:phenovalue[i])]

for (p in 1:length(randompheno)) {

newcase[randompheno[p],]<-phenodata[p,]

}

newped<-rbind(original.control,newcase)

filename<-paste("newcase-control_pheno",phenovalue[i],".ped",sep="")

write.table(newped,file=filename,col.names=F,row.names=F,quote=F,sep=" ")

}
