# Supplementary material for: The Impact of Phenocopy on the Genetic Analysis of Complex Traits
Source: PLoS One. 2010 Jul 29;5(7):e11876. doi: 10.1371/journal.pone.0011876 (PMC2912380; doi:10.1371/journal.pone.0011876)
Supplement: References S1 — References cited in the Supplementary Information. (0.03 MB DOC) [file pone.0011876.s003.doc]

**References:**

1. Schmidt M, Hauser ER, Martin ER, Schmidt S (2005) Extension of the SIMLA package for generating pedigrees with complex inheritance patterns: environmental covariates, gene-gene and gene-environment interaction. Stat Appl Genet Mol Biol 4: Article15.

2. Culverhouse R, Suarez BK, Lin J, Reich T (2002) A perspective on epistasis: limits of models displaying no main effect. Am J Hum Genet 70: 461-471.

3. Moore JH, Hahn LW, Ritchie MD, Thornton TA, White BC (2004) Routine discovery of complex genetic models using genetic algorithms. Applied Soft Computing 4: 79-86.

4. Pattin KA, White BC, Barney N, Gui J, Nelson HH, et al. (2009) A computationally efficient hypothesis testing method for epistasis analysis using multifactor dimensionality reduction. Genet Epidemiol 33: 87-94.
