# Supplementary material for: The Impact of Phenocopy on the Genetic Analysis of Complex Traits
Source: PLoS One. 2010 Jul 29;5(7):e11876. doi: 10.1371/journal.pone.0011876 (PMC2912380; doi:10.1371/journal.pone.0011876)
Supplement: Table S1 — The table summarizes the 10 best models for each phenocopy level identified during the MDR analysis. It has to be stressed that the MDR analysis has been conducted by performing 5.000 evaluations of possible interactions. An exhaustive analysis as implemented in the software would be computationally very intensive, as pointed out by the authors in a recent paper (see Pattin K. A. et al. [4]). In bold the correct SNPs as modelled in the purely epistatic penetrance function. (0.08 MB DOC) [file pone.0011876.s004.doc]

**Supplementary Table S1 – MDR interaction discovery**

| **Interaction** | **Testing accuracy** | | **Interaction** | | **Testing accuracy** |
| --- | --- | --- | --- | --- | --- |
|  |  |  | |  |  |
| **Phenocopy level 0%** | |  | | **Phenocopy level 20%** | |
| RL0-69_T,RL0-140_T,RL0-275_T | 0.62684447 |  | | RL0-69_T,RL0-140_T,RL0-275_T | 0.6084863 |
| **RL0-75**_T,RL0-149_T,**RL0-272_T** | 0.6079611 |  | | **RL0-75**_T,RL0-149_T,**RL0-272**_T | 0.59119713 |
| RL0-74_T,RL0-140_T,RL0-271_A | 0.59245 |  | | RL0-73_A,**RL0-153**_A,RL0-276_A | 0.5776614 |
| RL0-73_A**,RL0-153**_A,RL0-276_A | 0.5904 |  | | RL0-74_T,RL0-140_T,RL0-271_A | 0.5771996 |
| RL0-69_T,**RL0-153_A**,RL0-315_A | 0.58716106 |  | | RL0-69_T,**RL0-153**_A,RL0-315_A | 0.5755807 |
| RL0-14_A,**RL0-75**_T,RL0-140_T | 0.58657223 |  | | RL0-14_A,**RL0-75**_T,RL0-140_T | 0.57500523 |
| **RL0-75**_T,RL0-140_T,RL0-383_T | 0.5865667 |  | | RL0-69_T,**RL0-153**_A,RL0-301_0 | 0.5744562 |
| RL0-69_T,**RL0-153**_A,RL0-301_0 | 0.58638334 |  | | RL0-69_T,**RL0-153**_A,RL0-350_0 | 0.5744562 |
| RL0-69_T,**RL0-153**_A,RL0-350_0 | 0.58638334 |  | | RL0-69_T,RL0-140_T,RL0-324_T | 0.57419574 |
| RL0-69_T,RL0-140_T,RL0-330_T | 0.5861222 |  | | **RL0-75**_T,RL0-140_T,RL0-383_T | 0.5739757 |
| RL0-69_T,RL0-140_T,RL0-324_T | 0.58610004 |  | | **RL0-75**_T,RL0-140_T,RL0-381_A | 0.57380164 |
| **RL0-75**_T,RL0-140_T,RL0-381_A | 0.5860389 |  | | RL0-69_T,RL0-140_T,RL0-178_A | 0.5734935 |
| RL0-69_T,RL0-140_T,RL0-178_A | 0.58564997 |  | | RL0-69_T,RL0-140_T,RL0-330_T | 0.5732614 |
| RL0-79_T,**RL0-153**_A,RL0-175_A | 0.5851222 |  | | RL0-79_T,**RL0-153**_A,RL0-175_A | 0.5730044 |
| RL0-79_T,**RL0-153**_A,RL0-205_A | 0.5851 |  | | RL0-79_T,**RL0-153**_A,RL0-205_A | 0.5721812 |
| RL0-77_A,**RL0-153**_A,RL0-287_A | 0.5839722 |  | | RL0-69_T,RL0-83_T,**RL0-272**_T | 0.5717089 |
| RL0-69_T,RL0-113_T,**RL0-272**_T | 0.5837 |  | | RL0-77_A,**RL0-153**_A,RL0-287_A | 0.5715357 |
| RL0-75_T,RL0-115_T,**RL0-272**_T | 0.5837 |  | | RL0-79_T,RL0-89_T,**RL0-153**_A | 0.5714624 |
| RL0-79_T,RL0-89_T,**RL0-153**_A | 0.5834389 |  | | **RL0-75**_T,RL0-268_A,RL0-377_T | 0.571432 |
| RL0-4_A,RL0-69_T,RL0-270_T | 0.5832333 |  | | **RL0-75**_T,**RL0-272**_T,RL0-326_T | 0.5712869 |
|  |  |  | |  |  |
| **Phenocopy level 5%** | |  | | **Phenocopy level 30%** | |
| RL0-78_A,RL0-140_T,**RL0-272**_T | 0.6295997 |  | | RL0-69_T,RL0-140_T,RL0-275_T | 0.59896874 |
| RL0-69_T,RL0-140_T,RL0-275_T | 0.6224214 |  | | **RL0-75**_T,RL0-149_T,RL0-272_T | 0.5860952 |
| **RL0-75**_T,RL0-149_T,**RL0-272**_T | 0.6034261 |  | | RL0-74_T,RL0-140_T,RL0-271_A | 0.5712868 |
| **RL0-75**_T,**RL0-153**_A,RL0-283_T | 0.5909842 |  | | RL0-69_T,RL0-140_T,RL0-324_T | 0.570048 |
| RL0-74_T,RL0-140_T,RL0-271_A | 0.58910173 |  | | RL0-73_A,**RL0-153**_A,RL0-276_A | 0.56995153 |
| RL0-73_A,**RL0-153**_A,RL0-276_A | 0.58703995 |  | | RL0-14_A,**RL0-75**_T,RL0-140_T | 0.5694646 |
| RL0-69_T,**RL0-153**_A,RL0-315_A | 0.5850467 |  | | RL0-69_T,**RL0-153**_A,RL0-315_A | 0.5691747 |
| **RL0-75**_T,RL0-140_T,RL0-383_T | 0.5840283 |  | | **RL0-75**_T,RL0-140_T,RL0-381_A | 0.5691213 |
| RL0-69_T,**RL0-153**_A,RL0-301_0 | 0.5839182 |  | | RL0-69_T,**RL0-153**_A,RL0-301_0 | 0.5686533 |
| RL0-69_T,**RL0-153**_A,RL0-350_0 | 0.5839182 |  | | RL0-69_T,**RL0-153**_A,RL0-350_0 | 0.5686533 |
| RL0-14_A,**RL0-75**_T,RL0-140_T | 0.58369243 |  | | RL0-69_T,RL0-140_T,RL0-178_A | 0.56800437 |
| **RL0-75**_T,RL0-140_T,RL0-381_A | 0.5835299 |  | | **RL0-75**_T,RL0-140_T,RL0-383_T | 0.5678794 |
| RL0-69_T,RL0-140_T,RL0-178_A | 0.58343416 |  | | RL0-69_T,RL0-140_T,RL0-330_T | 0.56772804 |
| RL0-61_A,RL0-69_T,RL0-140_T | 0.583387 |  | | **RL0-75**_T,**RL0-272**_T,RL0-326_T | 0.56753224 |
| RL0-69_T,RL0-140_T,RL0-330_T | 0.58332044 |  | | RL0-79_T,**RL0-153**_A,RL0-175_A | 0.5669553 |
| RL0-69_T,RL0-140_T,RL0-324_T | 0.5828903 |  | | RL0-79_T,**RL0-153**_A,RL0-205_A | 0.566769 |
| RL0-79_T,**RL0-153**_A,RL0-175_A | 0.5821296 |  | | RL0-69_T,RL0-83_T,**RL0-272**_T | 0.5666441 |
| RL0-79_T,**RL0-153**_A,RL0-205_A | 0.5818472 |  | | RL0-69_T,RL0-113_T,**RL0-272**_T | 0.5663301 |
| RL0-20_T,**RL0-75**_T,RL0-140_T | 0.58176875 |  | | **RL0-75**_T,RL0-115_T,**RL0-272**_T | 0.5663301 |
| RL0-79_T,RL0-92_T,RL0-140_T | 0.58090687 |  | | **RL0-75**_T,RL0-175_A,RL0-268_A | 0.5661286 |
|  |  |  | |  |  |
| **Phenocopy level 10%** | |  | | **Phenocopy level 45%** | |
| RL0-78_A,RL0-140_T,**RL0-272**_T | 0.62510055 |  | | RL0-78_A,RL0-140_T,**RL0-272**_T | 0.58661586 |
| RL0-69_T,RL0-140_T,RL0-275_T | 0.6173962 |  | | RL0-69_T,RL0-140_T,RL0-275_T | 0.5847639 |
| **RL0-75**_T,RL0-149_T,RL0-272_T | 0.601388 |  | | RL0-78_A,RL0-148_A,RL0-223_A | 0.5840925 |
| **RL0-75**_T,**RL0-153**_A,RL0-283_T | 0.5874113 |  | | RL0-27_T,RL0-148_A,RL0-223_A | 0.57946813 |
| RL0-74_T,RL0-140_T,RL0-271_A | 0.58569825 |  | | RL0-70_T,RL0-135_T,RL0-226_A | 0.57936907 |
| RL0-73_A,**RL0-153**_A,RL0-276_A | 0.5845413 |  | | RL0-226_A,RL0-287_A,RL0-346_A | 0.57926726 |
| RL0-69_T,**RL0-153**_A,RL0-315_A | 0.5805399 |  | | RL0-226_A,RL0-289_A,RL0-346_A | 0.57926726 |
| RL0-69_T,**RL0-153**_A,RL0-391_A | 0.5804235 |  | | RL0-7_A,RL0-226_A,RL0-400_T | 0.57922035 |
| RL0-69_T,**RL0-153**_A,RL0-301_0 | 0.58024985 |  | | RL0-104_A,RL0-226_A,RL0-260_A | 0.5790413 |
| RL0-69_T,**RL0-153**_A,RL0-350_0 | 0.58024985 |  | | RL0-109_A,RL0-226_A,RL0-399_A | 0.57901317 |
| RL0-14_A,**RL0-75**_T,RL0-140_T | 0.5800257 |  | | RL0-1_A,RL0-226_A,RL0-282_T | 0.5789977 |
| **RL0-75**_T,RL0-140_T,RL0-381_A | 0.57974464 |  | | RL0-109_A,RL0-226_A,RL0-321_T | 0.57886404 |
| **RL0-75**_T,RL0-140_T,RL0-383_T | 0.5795736 |  | | RL0-148_A,RL0-223_A,RL0-354_A | 0.5788562 |
| RL0-69_T,RL0-140_T,RL0-330_T | 0.5795665 |  | | RL0-109_A,RL0-121_T,RL0-223_A | 0.57885617 |
| RL0-4_A,RL0-69_T,RL0-270_T | 0.5794619 |  | | RL0-197_T,RL0-226_A,RL0-354_A | 0.5788444 |
| RL0-4_A,RL0-69_T,**RL0-272**_T | 0.5794619 |  | | RL0-13_A,RL0-223_A,RL0-306_A | 0.57880616 |
| RL0-69_T,RL0-140_T,RL0-324_T | 0.5793917 |  | | RL0-11_A,RL0-148_A,RL0-226_A | 0.5787692 |
| RL0-69_T,RL0-140_T,RL0-178_A | 0.5791674 |  | | RL0-123_A,RL0-223_A,RL0-327_A | 0.5787625 |
| **RL0-75**_T,**RL0-272**_T,RL0-326_T | 0.5788002 |  | | RL0-51_T,RL0-66_A,RL0-226_A | 0.5787572 |
| RL0-61_A,RL0-69_T,RL0-140_T | 0.5787952 |  | | RL0-226_A,RL0-358_A,RL0-398_A | 0.5787467 |
